# Annotations

**Published:** 1892-12-17

**Authors:** 


### Annotations.

**Ophthalmia at Hanwell.**

The advantages of special knowledge and special experience are very clearly brought out by the "Report on Opthalmia," just published by Mr. Sydney Stephenson, Medical Officer to the Hanwell Ophthalmic School. The Pauper Schools for the Central London District are situated at Hanwell, and they have long been notorious for the excessive prevalence of ophthalmia among the inmates. So much was this the case, that in former years medical newspapers, The Hospital among the number, felt bound to protest very energetically against the inefficient means of isolation provided, and the consequent unnecessary suffering and injury of hundreds of poor children. All this is now changed. The change has been effected by the simple but obvious expedients of complete isolation and competent medical treatment: in a word, by dealing with the subject on the lines of common-sense and scientific thoroughness. We emphasise these points because evils like those which formerly prevailed at Hanwell now prevail elsewhere, and they are to be cured by methods exactly the same. The authorities at Hanwell, after long delay, woke to their duties with praiseworthy energy. They erected a magnificent ophthalmic school, that is, a school where all the sufferers from ophthalmia could be completely separated from the other children and yet continue their school work. They spent £30,000 on the buildings, and provided for 400 children. So successful has been the result that the hospital is now three times larger than the requirements of the patients, and it is suggested by Mr. Stephenson in his report that ophthalmic children from other pauper schools than those of the Central London District shall be sent to Hanwell for isolation and treatment: a most excellent suggestion, which we hope, in the interests of the ratepayers, to see promptly carried out. All these practically important changes have been wrought in less than four years. The change in the one year, from June, 1891, to June, 1892, is strikingly significant. At the beginning of the year there were 772 children in the schools, and 223 cases of ophthalmia; at the close there were 787 children in the school, but only 101 cases of ophthalmia. The managers and Dr. Sydney Stephenson are alike to be congratulated on these excellent results.

**Milk Pure v. Milk Skimmed.**

If people did but know the real value of milk, they would take a great deal of trouble to secure it good. A friend of the writer's is an extensive breeder of "short horns." He is constantly carrying off prizes at the agricultural shows for heifers and young bulls. What is the secret of his success? There are two secrets—heredity and milk feeding. Until his young animals are almost full grown he feeds them largely on milk. But what kind of milk does he give them? He gives them new milk; milk fresh from the cow; milk that has never seen a "skimmer"; milk that has never made the acquaintance of a "separator." Between milk as it flows from the cow's udder and "skimmed" or "separated" milk there is all the difference in the world. The cow yields milk; the skimmer and separator give water and salts, but specially water. Milk is milk by virtue of two things in particular, viz., its fat and its casein—that is, its contained butter and cheese. Skimmed milk is milk which has been deprived of practically all its fat and most of its rich casein. "Whangby" is the name of a cheese made in the North of England from skimmed milk. Have you ever tried to eat "Whangby," gentle sir, or madam? If you have, no further argument need be pursued with you. The obvious use of "Whangby" is, or was, for football purposes. It used to be given to farm labourers in harvest time. Their contempt for it was much too strong for words; they promptly threw it to the dogs, and the dogs, with most magnificent and disdainful sniffs, would leave it lying just where it had fallen. That sight the writer has many a time seen with his own eyes. "Whangby" is simply the cheese of "skimmed" milk. However poor "Whangby" may be, skimmed and separated milks are just as poor. As the dogs do with "Whangby," so would babies do with "skimmed" or "separated" milk if they were wise enough. The market, it is said, is full of "skimmed" and "separated" milks, in the "condensed" form. Now there is no objection to "condensed milk" provided it is "milk." But milk deprived of its fat and butter is not milk. That sounds rather Hibernian, but it is strictly true. Such milk is a fraud, and a wicked and cruel one. It is a fraud upon helpless babies, growing children, and sick persons of all ages. Do not buy "skimmed" or "separated" milk on any terms. Buy milk, either fresh or condensed, exactly as it was drawn from the cow, and if it be too rich you can "water" it down for yourself. We are informed, on the authority of Dr. Bernard Dyer, F.I.C., F.C.S., Secretary to the Society of Public Analysts, that "condensed milk" of the "Milkmaid" brand contains 11·95 per cent. of fat; that is to say, it consists of pure, unskimmed, unseparated milk. If that be indeed so, and we can hardly doubt it on such very unexceptionable authority, purchasers of condensed milk may congratulate themselves that there is at least one brand which is honestly what it professes to be, and therefore of full value for money, and excellent for all domestic purposes.

**Medical Watchmen and Cholera.**

"In time of peace prepare for war." London and the whole country will warmly approve of the Cholera Conference of Medical Officers of Health which commences its sittings at the Mansion House to-day, at the instigation of Dr. Collingridge, and will be very grateful to the Lord Mayor for his judicious and timely help. The Lord Mayor is a man of unusual intelligence and scientific culture, and it is pretty certain that under his presidency good practical work of an anticipatory and preventive kind will be effected. The subjects for discussion, which have been duly notified to all medical officers of health and to the Metropolitan Press, special and general, are of an entirely practical character. What are the actually effectual things which can be done at our ports to prevent cholera from getting a footing on our shores? This may be the most important of all national questions before we are five months older. It is a question we want asked and answered now. April and May will be the time for work, not for discussion. It is highly probable that cholera will make a determined attempt to invade us in the spring. We ought to act as if the threatened invasion were not merely probable, but certain. The medical officers of health of the kingdom are really so acting. Therein they are showing that the practical science of the times has taken a firm hold upon their intelligence, their imagination, and their resolution. The chief points which the Conference should consider are the questions of the "powers" they already possess by statute, and the "powers" they really require in order to deal effectually with the vessels, their passengers and their cargoes, which enter our ports. It is certain that the limited powers already given to medical officers of health will be found of little avail in the event of a determined invasion of cholera. The most practical thing the Conference can do is to formulate a Bill of a temporary character, which shall confer upon them adequate powers, and which shall be of such a reasonable nature as to commend itself to Parliament and the public. In this somewhat difficult task we wish the Conference all possible success.

